# Magnetically Recoverable Magnetite/Gold Catalyst Stabilized by Poly(*N*-vinyl-2-pyrrolidone) for Aerobic Oxidation of Alcohols 

**DOI:** 10.3390/molecules16010149

**Published:** 2010-12-29

**Authors:** Hsiao Wei Chen, Arumugam Murugadoss, T.S.Andy Hor, Hidehiro Sakurai

**Affiliations:** 1 Department of Chemistry, National University of Singapore, 3 Science Drive 3, 117543, Singapore; E-Mails: g0300251@nus.edu.sg (H.W.C.); andyhor@nus.edu.sg (T.S.A.H.); 2 Research Center for Molecular Scale Nanoscience, Institute for Molecular Science, Myodaiji, Okazaki 444-8787, Japan; E-Mail: ammuruga@ims.ac.jp (A.M.); 3 Institute of Materials Research and Engineering, Agency for Science, Technology and Research, 3 Research Link, S117602, Singapore; 4 PRESTO, Japan Science and Technology Agency, Tokyo 102-0075, Japan

**Keywords:** poly(*N*-vinylpyrrolidone), magnetite, gold nanoclusters, alcohol oxidation, green chemistry

## Abstract

Fe_3_O_4_:PVP/Au nanocomposite synthesized via a two-step procedure was tested as a quasi-homogenous alcohol oxidation catalyst. It was found that the nanocomposite was able to carry out aerobic oxidation of alcohols in water at room temperature. Studies show rapid magnetic recoverability and reusability characteristics.

## 1. Introduction

In recent years, energy has become an important concern, leading to the desire for better, more efficient catalysts to replace current industrial processes [1,2]. These days, sustainability and environmental friendliness are key elements to consider in the realm of catalysis. While catalysis is an important process for the development of starting materials, fine chemicals and pharmaceuticals, green catalysis requires the synthesis of environmentally benign catalysts which can be easily removed from the reaction media and recycled many times with very high efficiency [3].

Since Haruta’s breakthrough discovery of oxide-supported Au nanocluster (Au NC) catalysts for CO oxidation at low temperatures [4,5], interest in gold chemistry in the field of catalysis has grown exponentially [6,7,8,9]. Gold has been shown to be capable catalyzing many organic transformations, some of them uniquely [10,11,12,13,14,15,16], due to the relativistic effect of the element [17] that allows for new modes of catalysis. This meteoric rise in popularity in gold catalysts is reflected in the recent reviews on the trend and future direction of this research field [7,8,9,18,19] and on the recent use of gold catalysts in an atom-economic and chemoselective total synthesis of bryostatin 16 [20].

Au NCs of 1.3 nm to 2 nm in diameter protected by poly(*N*-vinyl-2-pyrrolidone) (PVP) have been proven to perform aerobic oxidation of alcohols at ambient temperatures [21,22]. Other metal nanoclusters on magnetite have recently been reported to be active, magnetically recoverable and reusable [23,24]. Notably, a nano-ferrite-Pd catalyst [23] has been used in the oxidation of alcohols as well, albeit using H_2_O_2_ rather than molecular oxygen as the oxidant. This is encouraging as it indicates that the use of nano-ferrite as the support does not deactivate the supported catalyst towards oxidation catalysis, and the choice of oxidant can be further improved by using molecular oxygen, or even air.

Au NCs have also been successfully attached onto magnetite either in dumbbell-shape fashion [25,26,27] or as a core-shell motif [28,29,30]. It has further been found that such Au NCs on iron oxides can perform aerobic oxidation of amines either as heterogenous catalysts [31,32], or as CO oxidation catalysts [25].

PVP-supported iron oxides are known in the literature [33,34,35], and have also been found to have good dispersity [35], which is potentially important in maintaining the “quasi-homogeneous” property of the composite in relation to the parent Au:PVP system [21,22,36,37,38,39,40,41,42,43,44,45] which has already been established.

Although no clear mechanism has been defined for the oxidation process [46], it has been determined that the size of gold on the surface is not the key issue, but rather the nature of interface [47] and the presence of oxygen in the form of oxides [48].

While there has been some work done with Au NCs stabilized by PVP, Fe_3_O_4_ stabilized by PVP and Au nanoparticles on Fe_3_O_4_, there is a lack of information where all three elements are present, especially in the field of alcohol oxidation. Our work here aims to address this gap, although the concept of using magnetic nanoparticles as *quasi*-homogenous catalysis supports has been known [49]. Herein, we describe our attempt to make magnetically recoverable and reusable quasi-homogenous Fe_3_O_4_/Au PVP-stabilized catalyst for the aerobic oxidation of alcohols.

## 2. Results and Discussion

### 2.1. Catalyst Preparation

The catalyst was prepared by a two-step wet chemical process, whereby the magnetite support stabilized by PVP [50] was first isolated, followed by the addition and reduction of the gold precursor to afford the desired composite catalyst. The magnetite particles were synthesized via a modified literature method [43,51,52], by reduction of a mixture of Fe^2+^ and Fe^3+^ ions [51,52] using ammonia solution in the presence of the PVP polymer. Our novel one-pot synthesis is more environmentally friendly than earlier reports, as it can be carried out at lower temperatures [34,35], without the use of organic solvents and surfactants [53,54,55]. In most cases, a much stronger base like sodium hydroxide [35] is used for the reduction of iron salts to the magnetite core was well, and our switch to ammonia as a reductant addresses the waste disposal problems associated with this process.

**Figure 1:**
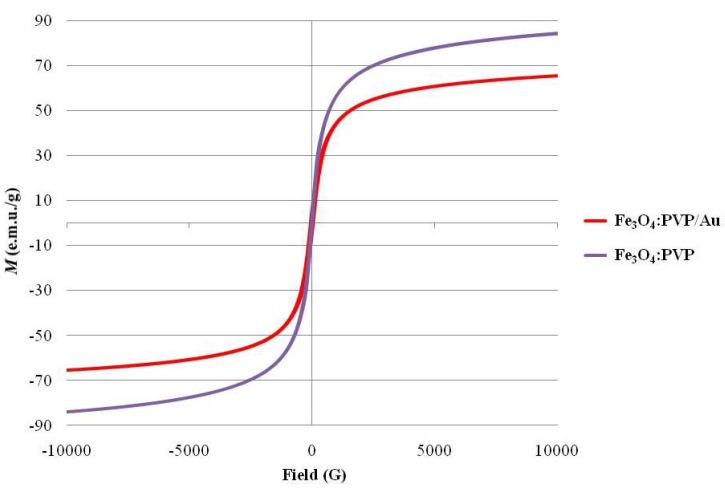
VSM data for the **Fe_3_O_4_:PVP** support and the **Fe_3_O_4_:PVP/Au** composite, conducted at 100K.

Magnetic recoverability is one main benefit which led to the development of this system, and as such, the Vibrating Sample Magnetometry (VSM) measurements are of great interest. VSM studies of the support and the composite (Figure 1) show hysteresis loops typical for superparamagnetic particles [56]. The composite **Fe_3_O_4_:PVP/Au** is less magnetic than its corresponding **Fe_3_O_4_:PVP** support, which is unsurprising, given the shielding effect of having gold on the outside of the PVP-stabilized magnetite. With regard to other known synthetic magnetic materials [35,55,56], the **Fe_3_O_4_:PVP/Au** and the **Fe_3_O_4_:PVP** support both show sufficient superparamagnetic behavior, as exhibited by strong magnetic moments of 65.6 and 84.4 e.m.u./g, respectively.

**Figure 2:**
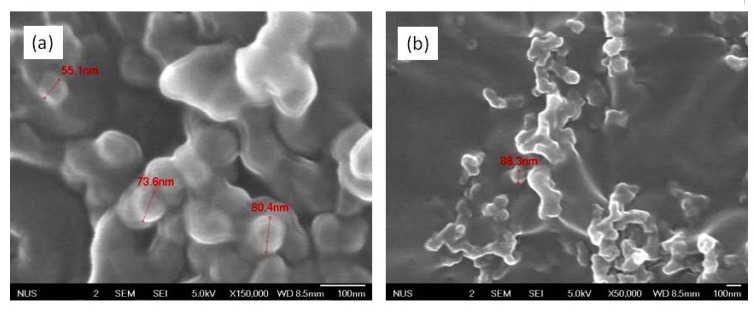
**(a)** FESEM image of the **Fe_3_O_4_:PVP** support at 150,000 times magnification. **(b)** FESEM image of the **Fe_3_O_4_:PVP/Au** composite at 50,000 times magnification.

Field Emission Scanning Electron Microscope (FESEM) images of **Fe_3_O_4_:PVP/Au** prepared at 275K show large particle sizes [Figure 2(b)] of approximately 80 nm in diameter, which is unsurprising due to the longer chain of the PVP stabilizer used. In this case, PVP(K-90) [50] with an average molecular weight of 360,000 was used, in lieu of the PVP(K-30) of molecular weight 30,000 in the case of **Au:PVP** [43]. The longer chain of the PVP polymer is evident in the encapsulation layer visible around the particles. A slight, expected increase in the particle size is observed for the composite [Figure 2(b)] compared to the support alone [Figure 2(a)].

**Figure 3:**
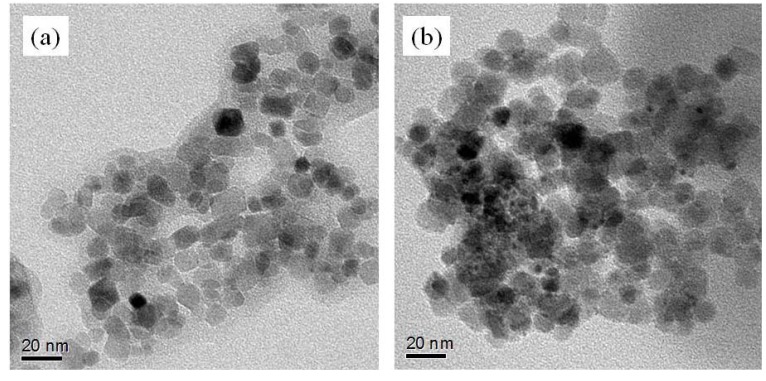
**(a)** TEM image of the **Fe_3_O_4_:PVP** support. **(b)** TEM image of the **Fe_3_O_4_:PVP/Au** composite.

Transmission Electron Microscopy (TEM) images of the **Fe_3_O_4_:PVP/Au** composite [Figure 3(b)] shows inhomogeneity in the coating of Au NCs on the iron support. This is probably because even at low temperatures of 275K, there exists the possibility of the HAuCl_4_ to be instantaneously reduced by the Fe^2+^ present within the iron support should the two substances come into contact. Such reductions without the need for a reducing agent are known in the literature [57]. Other Au atoms were probably first pre-coordinated to the PVP before addition of NaBH_4_ give **Au:PVP** clusters similar to those of the original system [21,22,36,37,38,39,40,41,42,43,44,45,58]. TEM studies show very few small Au NCs, which is unexpected based on the earlier reported **Au:PVP** systems [21,22].

**Figure 4:**
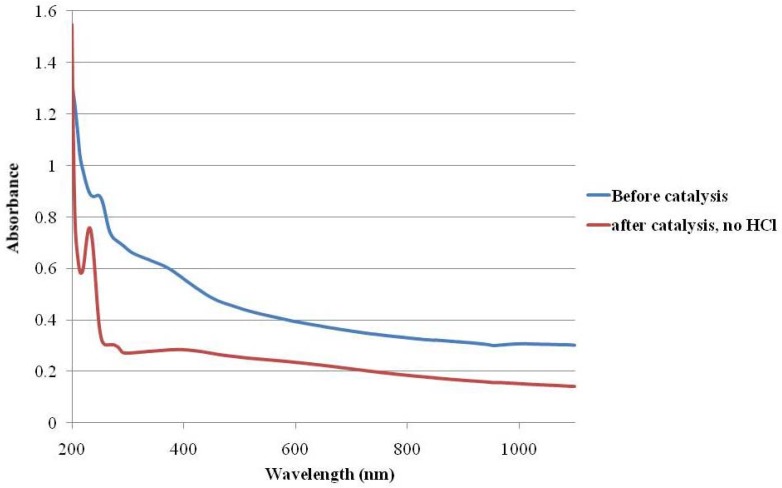
UV-Vis Spectra for **Fe_3_O_4_:PVP/Au** in milliQ water, both before and after the alcohol oxidation, conducted at 300K.

It was, however, decided to carry on with the catalytic screening based on the absence of a gold Surface Plasmon Resonance (SPR) band around the 520 nm region [59] in the UV-Visible spectrum for this composite. (Figure 4) The presence of such a band would indicate the existence of large nanoclusters of gold in the catalyst, which inevitably give rise to poorer activities [22].

### 2.2. Catalytic Screening and Substrate Scope

**Fe_3_O_4_:PVP/Au** was screened against various substrates in aerobic alcohol oxidation. All the reactions were carried out using 2 atom% (per Au) of **Fe_3_O_4_:PVP/Au**, in the presence of 300 mol% of K_2_CO_3_ in water under ambient conditions. The results are summarized in Table 1 with the yields with **Au:PVP** catalyst under the same conditions in parentheses for reference. In the reaction with benzyl alcohol, aerobic oxidation took place giving the mixture of benzaldehyde and benzoic acid in 5% and 94% yields, respectively (Entry 1). Although the reactivity of **Fe_3_O_4_:PVP/Au** was slightly different from that of **Au:PVP**, which yielded benzoic acid selectively in 98% yield, the **Fe_3_O_4_:PVP/Au** catalyst was found to be of sufficient catalytic activity to monitor with other benzylic alcohols.

As shown in Entry 2, when screened against *para*-hydroxybenzyl alcohol, the corresponding aldehyde and acid were obtained in 42% and 30% yields, respectively, and a considerable amount of polymers were produced, which was contrary to the result with **Au:PVP** which gave the selective formation of aldehyde in 91% yield [60]. The **Fe_3_O_4_:PVP/Au** composite also gave the aldehyde as a major product in the reaction with *para*-methoxybenzyl alcohol (*entry 3*) in 42% yield, together with the acid in 32% yield. Under the same reaction conditions, exclusive formation of the acid (93% yield) was observed in the **Au:PVP**-catalyzed reaction.

In contrast, **Fe_3_O_4_:PVP/Au** exhibited comparable reactivity to **Au:PVP** in the reaction with the substrates with electron-withdrawing group such as *para*-nitrobenzyl alcohol (*entry 4*) or cyclic benzyl alcohol such as 1-indanol (Entry 5). In both cases, quantitative yield of the product was obtained under the same reaction conditions.

**Table 1:** Aerobic alcohol oxidation of various benzyl alcohols by **Fe_3_O_4_:PVP/Au**. Values in parentheses correspond to those for the established **Au:PVP** catalyst [39]. 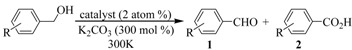

Entry	R	Time (h)	GC Yield (%)	
			1	2
1	H	6	5	94 (98)
2	*p*-OH	8	42^a^ (91)	30^a^ (8)
3	*p*-OMe	3	42	32 (93)
4	*p*-NO_2_	24	-	99 (99)
5	1-indanol^b^	0.5	98 (>99)	-

^a^ Isolated yield. The remainder of the substrate was polymerized. ^b^ Product is 1-indanone.

### 2.3. Recoverability and Reusability Characteristics

**Figure 5:**
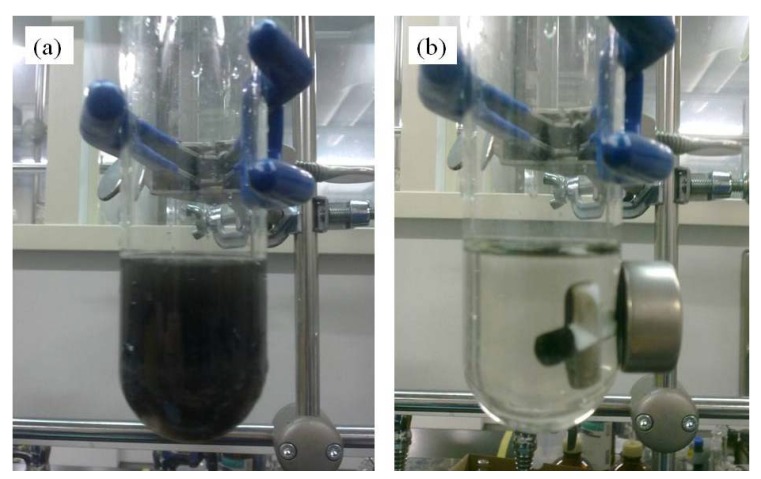
**(a)** The composite dispersed in water during the reaction. **(b)** Magnetic recovery of the composite after the reaction, using a strong external neodynium magnet.

The recoverability and reusability of the **Fe_3_O_4_:PVP/Au** catalyst is one of its key features. The ease of recoverability is demonstrated in Figure 5. A typical duration for recovery of the **Fe_3_O_4_:PVP/Au** composite is 1 minute. The neodynium magnet used for retrieval has a pull force of over 35 pounds, much more than the usual magnetic stirring bar. Hence, during the reaction, the catalyst remains dispersed in solution as seen in Figure 5(a), even for the catalysis involving *para*-nitrobenzyl alcohol which has a 24-hour reaction time. Vigorous stirring and the much weaker magnetic field of the stirring bar ensures that the monodispersity is maintained in solution.

The ability to recover the catalyst quickly gives us the option of first recovering the catalyst, followed by neutralization of the reaction mixture only. The neodynium magnet is applied to first magnetically separate the catalyst, and the reaction mixture decanted into another vessel, with washing, before addition of acid is done to neutralize the base used in the catalysis. This solves one of the key problems of the older **Au:PVP** system, *i.e.* the aggregation of gold in the presence of acid. InFigure 4, this is exhibited by a lack of growth of an SPR band at the region around 520 nm [59].

Stark and his co-workers reported that carbon-coated cobalt particles exhibit rapid magnetic separation ability due to the strong magnetic saturation [61]. In comparison with Stark’s example, the above **Fe_3_O_4_:PVP/Au** showed slower recovery. This is unsurprising, given the lower magnetic saturation values caused by the use of PVP, which forms a protective film around the magnetic core [Figure 2(a)]. In contrast, this protective film serves to protect the magnetite from oxidation during storage. The samples are stable for 1-2 months at room temperature without having to store them under nitrogen atmosphere.

**Table 2:** Recoverability studies for the aerobic oxidation of benzyl alcohol by **Fe_3_O_4_:PVP/Au**. 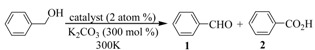

Entry	Cycle	GC Yield (%)		Conversion (%)
		1	2	
1	1	5	94	99
2	*2*	6	92	98
3	*3*	8	90	98
4	*4*	8	89	97
5	5	6	88	94

To prove the magnetic recoverability and reusability of the composite, the catalyst was used to oxidize simple benzyl alcohol for 5 cycles. Under the standard conditions of benzyl alcohol oxidation with 2 atom% (per Au) of **Fe_3_O_4_:PVP/Au**, in the presence of 300 mol% of K_2_CO_3_ in water under ambient conditions for six hours, it can be concluded that **Fe_3_O_4_:PVP/Au** possesses satisfactory reusability although conversion yield was slightly decreased to 94% after 5 cycles (Table 2). Like other examples [62], the magnetite cores are fairly stable under the reaction conditions, therefore the slight decrease in conversion yield might be due to the aggregation of gold clusters. It is noteworthy that **Au:PVP** catalysts [39] could not be reused in the reaction with benzyl alcohol because the aggregation of the catalyst by benzoic acid, the product, was unavoidable, causing the drastic deactivation of the catalyst. While the star polymer variant of the system with LCST-type thermosensitiveness [45] gave superior reusability in the oxidation of benzyl alcohol to benzoic acid in >99% conversion over six cycles, the current magnetically recovery system might be easier in practical operation.

## 3. Experimental

### 3.1. General

The composites prepared were characterized by VSM, UV-visible (UV-vis) optical spectroscopy, FESEM, and TEM. VSM studies were performed on a LakeShore 7400 calibrated with a Ni Sphere Model 730908 with field applied with a 4-inch magnet across a gap of 1.5 inches. UV-vis absorption spectra were recorded at ambient temperature using a double-beam spectrophotometer (V-670, JASCO). FESEM studies were conducted on a JEOL 6701F Spectrometer. TEM images were recorded at an acceleration voltage of 100 kV (Hitachi H-7500) with a typical magnification of 100,000.

### 3.2. Preparation of Fe_3_O_4_:PVP Support

The **Fe_3_O_4_:PVP** support with various PVP chain lengths can be prepared by the rapid and simultaneous reduction of a FeCl_2_-FeCl_3_ (1:2) mixture by NH_4_OH in an aqueous solution of PVP at 353 K using Schlenk techniques. Specifically, PVP (K-90) (555.5 mg, 5 mmol in monomer units) was added to degassed milliQ water (10 mL) and stirred for 30 minutes at room temperature to obtain an aqueous solution. 2 M FeCl_2_ in 2 M HCl (125 μL, 0.25 mmol) and 1M FeCl_3_ in 1M HCl (500 μL, 0.5 mmol) were added and the resulting mixture was stirred slowly at ambient temperature for another 30 mins under inert atmosphere. The temperature was then raised to 353 K and the stirring rate increased. NH_4_OH (6.25 mL of 0.7 M solution diluted to 40 mL with degassed milliQ) was injected under inert conditions and with vigorous stirring. After a further 1 h of stirring, the resulting hydrosol of **Fe_3_O_4_:PVP** was purified via magnetic decantation three times using 20 mL of degassed milliQ. Finally, the black **Fe_3_O_4_:PVP** solid was dried *in vacuo* to obtain powders for easy storage.

### 3.3. Preparation of Fe_3_O_4_:PVP/Au Composites

The **Fe_3_O_4_:PVP/Au** composite was prepared by the rapid and simultaneous reduction of a HAuCl_4_ by NaBH_4_ in an quasi-homogenous solution of the respective **Fe_3_O_4_:PVP** supports at low temperature using Schlenk techniques. A quasi-homogeneous solution (25 mL) of **Fe_3_O_4_:PVP** (25.0 mg) was prepared by sonication for 45 mins in an ice bath under inert atmosphere. To the solution HAuCl_4_ (833.4 μL of 50 mM solution, 41.67 mmol) was added at 275 K under vigorous stirring. After stirring for 30 min at 275 K, the aqueous solution of NaBH_4_ (0.1 M, 2.5 mL) was rapidly injected into the mixture and further stirred for 1 h. The resulting hydrosol of **Fe_3_O_4_:PVP/Au** was dialyzed three times by magnetic decantation. Similarly, the black **Fe_3_O_4_:PVP/Au** solid was dried *in vacuo* to obtain powders for easy storage.

### 3.4. General Procedure for Aerobic Oxidation of Benzyl Alcohols

Aerobic oxidation of the alcohol substrate **1** to the corresponding aldehyde **2** was carried out using an organic synthesizer (EYELA, PPS-2510). Changes were made to the earlier process [39] to accommodate the magnetic recovery process, as the **Fe_3_O_4_:PVP** cores are unstable toward mineral acids. Benzyl alcohol (0.25 mmol), K_2_CO_3_ (103.6 mg, 0.75 mmol), and H_2_O (5 mL) were placed into a test tube (Φ = 30 mm). **Fe_3_O_4_:PVP/Au** (15.1 mg, 2 atom %) was first added to degassed milliQ water (10 mL) and sonicated for 45 minutes to obtain a quasi-homogenous solution. This solution was added to the reaction mixture and stirred vigorously at 300 K. The catalyst was separated magnetically using a commercial neodynium magnet for retrieval, and the reaction solution quenched with 1 M HCl (1.5 mL). The products were extracted with ethyl acetate (3x20 mL) and dried *in vacuo*. The residue was redisssolved in ethyl acetate (30 mL) and a portion (200 μL) of this crude mixture was added to ethyl acetate (1.5 mL) and 6 mM hexadecane (200 μL) as internal standard, to make up the solution analyzed using gas chromatography (Shimadzu, GC-2014). The GC yield was estimated from the calibration curve.

## 4. Conclusions

To date, this is the only known report on the use of PVP as a stabilizer to support both magnetite and gold nanoclusters to form a composite. The benefit to such a design is the ability to effect the magnetic recoverability of the magnetite and the aerobic catalytic oxidation ability of the nanoclusters while retaining the quasi-homogenous solubility of the parent **Au:PVP** catalyst. Such a quasi-homogenous catalyst has a larger surface area compared with heterogenous catalysts, as well as better potential in terms of substrate scope as the catalysis can be done in one phase.

In our preliminary catalytic studies, we have found that Fe_3_O_4_/Au stabilized by PVP (K-90) show good activity, selectivity and recoverability, despite a lack of small Au NCs (~ 1.3 – 3 nm) seen in the TEM images. This points to two possibilities, one of which is that there are very small, sub-nm clusters responsible for the high activity, which are not visible in the TEM. Another is that the support so greatly enhances activity that it overcomes the size requirement of the gold clusters, similar to heterogenous CO oxidation catalysts.

Compared with our earlier systems, the magnetic recovery process possesses several benefits: one is that the reuse of the catalyst is much easier and more speedy. Another is that the recovery operated before the neutralization of the reaction media can prevent the significant aggregation of the catalyst, which is proceeded under acidic conditions and which causes deactivation of the catalyst.
